# Is cancer-related death associated with circadian rhythm?

**DOI:** 10.1186/s40880-019-0373-9

**Published:** 2019-05-14

**Authors:** Shing Fung Lee, Miguel Angel Luque-Fernandez

**Affiliations:** 1 Department of Clinical Oncology, Tuen Mun Hospital, New Territories West Cluster, Hong Kong, P. R. China; 2 Department of Non-Communicable Disease and Cancer Epidemiology, Biomedical Research Institute of Granada, University of Granada, 18002 Granada, Spain; 3 Department of Non-Communicable Disease and Epidemiology, London School of Hygiene and Tropical Medicine, London, WC1E 6HW UK; 4 Department of Epidemiology, Harvard School of Public Health, Boston, MA 02115 USA

Dear editor,

Biological periodicities occur based on a periodicity in a spectrum of frequencies ranging from milliseconds to years. A circadian rhythm is any biological process that displays an endogenous oscillation of about 24 h [1]. Circadian rhythm is the adaption of our bodies’ biological processes to the day and night cycle. The biological clock is reset daily to synchronize with the exposure to sunlight because our internal clock repeats at around 24 h [1]. Because of the use of artificial light, our internal clock is influenced by multiple factors, including social interactions [1].

Our bodily functions are maintained by coordination among varying variables, such as hormonal levels, body temperature, cognitive function, and sleep, that closely follow the circadian rhythm [1]. Multiple studies suggested that the temporal pattern of cardiac death is not uniform during the day [2]. Only a handful of studies have assessed the temporal pattern of death in cancer patients, two of which were conducted in palliative care units [3, 4], and one study included patients who died in other wards [5].

All these studies were conducted at single institutes with small sample sizes, and their statistical analyses involved multiple comparisons, which might have jeopardized the validity of the results. If a temporal pattern of cancer death is identified, healthcare service could be optimized to better support terminally ill patients and their family. Families can be counseled appropriately and be psychologically prepared for the final hours of patient’s life. Therefore, our goal was to investigate the temporal pattern of death in cancer patients using a large sample size and robust statistical methods to account for chronobiological periodicity.

The methods in the present study are detailed in the Additional file 1: Materials and methods. A total of 353,827 cases of death reported between January 2008 and December 2016 in public hospitals as in-patients in Hong Kong were identified: 58,451 (16.5%) died of cancer, 21,544 (6.1%) died of ischemic heart disease, and 91,999 (26.0%) died of pneumonia as main causes (Table 1). The observed number of cancer deaths was the most frequent at 6:00–6:59 am, having the prevalence ratio of 1.24 (95% confidence intervals [CI] 1.04–1.47). The highest prevalence ratios for deaths due to ischemic heart disease (1.49, 95% CI 1.20–1.85) and pneumonia (1.35, 95% CI 1.08–1.67) were both observed at 8:00–8:59 pm (Table 2, Additional file 2: Figure S1).

**Table 1:** Demographic information for the 171,994 death cases due to cancer, ischemic heart disease, and pneumonia in Hong Kong, 2008–2016

Characteristic	Death due to cancer	Death due to ischemic heart disease	Death due to pneumonia
Total (cases)	58,451	21,544	91,999
Age (years)			
Median (range)	70 (18–108)	82 (19–113)	85 (18–117)
Sex [cases (%)]			
Male	33,132 (56.7)	11,211 (52.0)	51,481 (56.0)
Female	25,319 (43.3)	10,333 (48.0)	40,518 (44.0)
Race [cases (%)]			
Chinese	57,185 (97.8)	20,716 (96.2)	89,911 (97.7)
Others or unknown	1266 (2.2)	828 (3.8)	2088 (2.3)

**Table 2:** Prevalence ratios of 171,994 deaths by hour per day, age, and sex in Hong Kong, 2008–2016

Time/age/sex	Prevalence ratio (95% CI)		
	Death due to cancer	Death due to ischemic heart disease	Death due to pneumonia
During 0:00–0:59 am	Reference	Reference	Reference
During 1:00–1:59 am	1.05 (0.88–1.26)	1.02 (0.82–1.26)	0.99 (0.79–1.24)
During 2:00–2:59 am	1.00 (0.84–1.19)	1.02 (0.81–1.27)	1.00 (0.80–1.26)
During 3:00–3:59 am	1.03 (0.86–1.23)	1.08 (0.86–1.34)	1.07 (0.86–1.35)
During 4:00–4:59 am	1.04 (0.87–1.24)	1.15 (0.93–1.43)	1.07 (0.85–1.34)
During 5:00–5:59 am	1.06 (0.89–1.26)	1.18 (0.95–1.46)	1.05 (0.84–1.31)
During 6:00–6:59 am	1.24 (1.04–1.47)	1.25 (1.00–1.55)	1.27 (1.01–1.58)
During 7:00–7:59 am	0.96 (0.81–1.15)	0.90 (0.72–1.12)	0.85 (0.68–1.07)
During 8:00–8:59 am	1.08 (0.91–1.29)	1.01 (0.81–1.26)	0.96 (0.77–1.20)
During 9:00–9:59 am	1.10 (0.93–1.31)	1.21 (0.97–1.50)	1.14 (0.91–1.43)
During 10:00–10:59 am	1.09 (0.92–1.30)	1.35 (1.09–1.68)	1.17 (0.93–1.46)
During 11:00–11:59 am	1.08 (0.91–1.28)	1.19 (0.96–1.48)	1.22 (0.97–1.52)
During 12:00–12:59 pm	1.18 (0.99–1.40)	1.17 (0.94–1.45)	1.21 (0.97–1.51)
During 1:00–1:59 pm	1.07 (0.90–1.27)	1.25 (1.01–1.56)	1.20 (0.96–1.51)
During 2:00–2:59 pm	1.09 (0.92–1.30)	1.06 (0.86–1.32)	1.14 (0.91–1.43)
During 3:00–3:59 pm	1.06 (0.89–1.26)	1.13 (0.91–1.40)	1.16 (0.92–1.45)
During 4:00–4:59 pm	1.18 (0.99–1.40)	1.14 (0.92–1.41)	1.24 (0.99–1.55)
During 5:00–5:59 pm	1.03 (0.87–1.23)	1.20 (0.97–1.49)	1.13 (0.91–1.42)
During 6:00–6:59 pm	0.96 (0.81–1.14)	0.93 (0.74–1.16)	1.01 (0.80–1.26)
During 7:00–7:59 pm	0.97 (0.82–1.16)	1.04 (0.84–1.29)	1.05 (0.84–1.31)
During 8:00–8:59 pm	1.22 (1.02–1.45)	1.49 (1.20–1.85)	1.35 (1.08–1.67)
During 9:00–9:59 pm	1.13 (0.95–1.34)	1.14 (0.92–1.42)	1.14 (0.91–1.43)
During 10:00–10:59 pm	1.03 (0.86–1.23)	1.25 (1.00–1.55)	1.12 (0.90–1.41)
During 11:00–11:59 pm	1.07 (0.90–1.27)	1.30 (1.05–1.61)	1.26 (1.00–1.58)
Other variables			
Sex (male versus female)	1.33 (1.26–1.40)	1.25 (1.17–1.33)	1.77 (1.66–1.90)
Age (per 1 year increment)	1.03 (1.02–1.03)	1.04 (1.04–1.04)	1.06 (1.06–1.06)

*CI* confidence interval

Figure 1 shows the observed temporal pattern of cancer deaths in hours (Fig. 1a) and minutes (Fig. 1b) of the day. Even if a small increase in the number of deaths at 6:00–6:59 am and 8:00–8:59 am was observed, there was no evidence of a unimodal sinusoidal circadian rhythm (periodicity) in the time of death according to the parametric sinusoidal circadian test (*Z* = 2.06, *P* = 0.127) and the non-parametric multimode test (H0: one unique mode, excess mass = 0.011, *P* < 0.001). The same analyses were repeated on deaths due to ischemic heart disease and pneumonia. Based on the circadian test, a unimodal circadian rhythm in the former, but not in the latter was observed (Additional file 3: Figure S2 and Additional file 4: Figure S3).

**Fig. 1:**
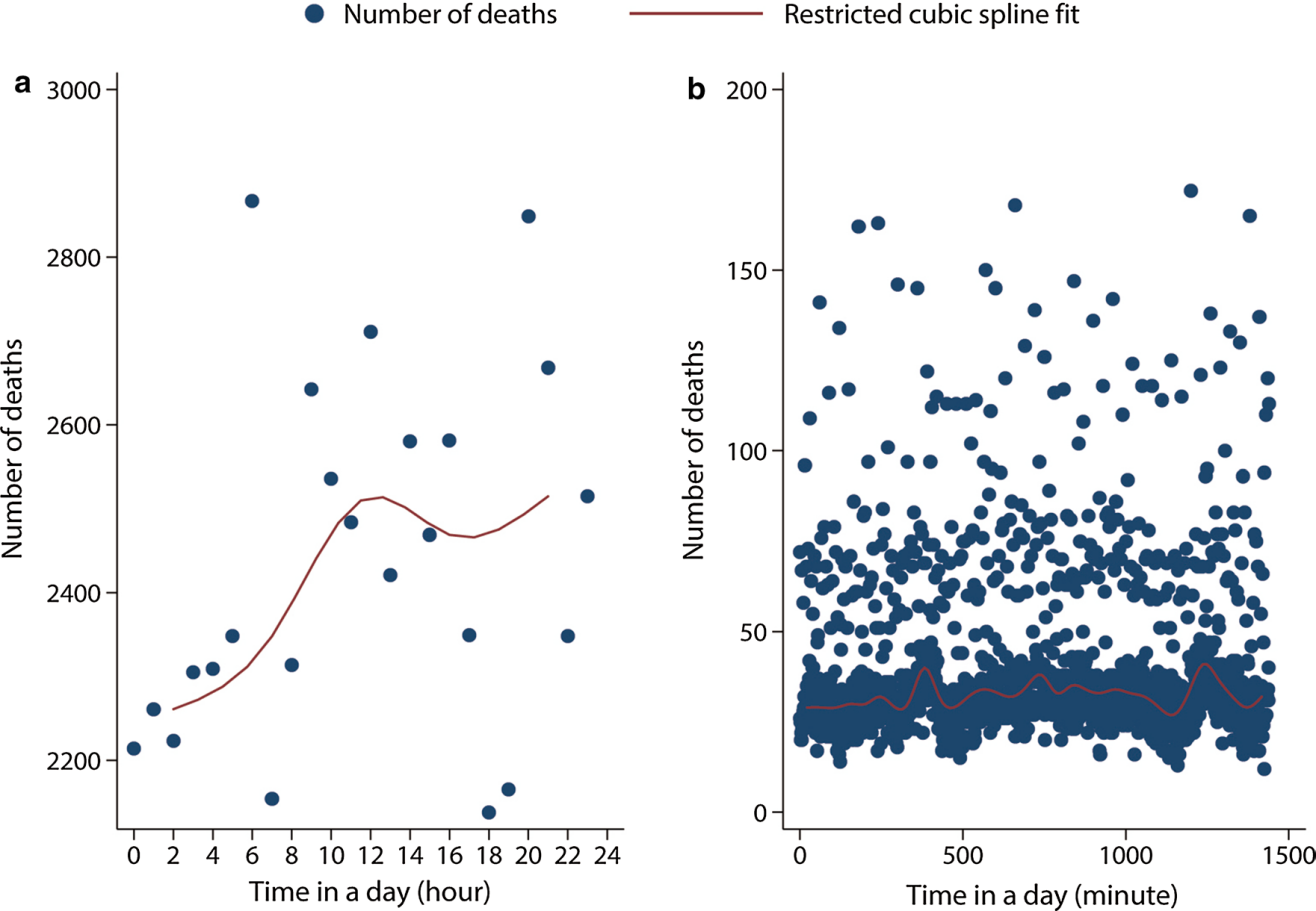
Scatter plot of the number of deaths by time in hours (**a**) and in minutes of the day (**b**), showing the temporal pattern of cancer deaths in acute oncology and cancer palliative wards in Hong Kong, 2008–2016. We found no evidence of a unimodal sinusoidal circadian rhythm (periodicity) in the time of cancer deaths according to the parametric sinusoidal circadian test (*Z* = 2.06, *P* = 0.127). Restricted cubic splines using three knots were fitted to model the number of deaths in each hour of the day. The resulting spline fit is graphed as a red line

We did not detect a circadian pattern of cancer death. The present study evaluated the temporal pattern of death among cancer patients using trigonometric functions and with time modeled in a circular scale. This robust method used to model periodicity validated the absence of circadian rhythms. Furthermore, we considered a large sample in hospital settings.

Results from the present study were in contrast with those of prior studies on the temporal pattern of death in cancer patients [3–5]. Neumann et al. [3] revealed a peak of cancer death between 8:00 am and 10:00 am in palliative care wards. In the study of Goncalves et al. [5], peaks of cancer death were observed in a public hospital’s palliative care ward between 0:00 am and 2:00 am and between 8:00 am and 10:00 am, with a trough at 4:00–8:00 am. Davies et al. [4] observed a trough in cancer death at 2:00–5:00 am, with no peak. Even if these previous studies on circadian rhythms of cancer death in other geographical areas and periods contradict our findings, the evidence was limited, and the methods used to identify circadian rhythms were not the most appropriate and robust. In contrast, our statistical approach, modeling time in a circular scale and testing parametrically for the presence of a circadian pattern, internally validated our findings. However, more evidence is needed in different populations and geographic areas to externally validate these observations.

A potential trigger of sudden cardiac events and death in the morning hours is related to the increase in catecholamine levels and blood viscosity when assuming a switch from a supine posture to an upright posture after waking up. However, hospitalized cancer patients in general are cachexic and are sicker than patients with many other types of disease, and the upright posture may be less likely to be a contributor to cancer death. Indeed, we did not identify a morning peak in cancer death, suggesting that cancer death is not mainly driven by the above mechanisms.

The body has the ability to synchronize internal biological processes with daily external events; the circadian timing system is responsible for this ability [6]. A critical function of the system is the organization and scheduling of behavioral and physiological events [6]. It influences the responsiveness to stress and challenges in different hours of the day. Disturbance in the rest-activity circadian rhythm is common in cancer patients [7]. The etiology of the disturbance is multifactorial; altered physiology related to the disease, symptoms, psychological stress, and cancer treatment all appear to play a role in disrupting circadian regulatory processes [8, 9]. The frequent disruption of the biological circadian rhythm in cancer patients is possibly associated with the lack of circadian patterns of death in our cohort. Another possible explanation may be the influence of factors related to routine practices in the wards, such as scheduling of medication and different levels of nursing activity and care, including bathing and dressing during a 24-h period. Furthermore, it is possible that incidental cancer deaths are more often registered at 6:00–6:59 am and at 8:00–8:59 pm because these periods coincide with a change between night and day shifts.

The present study has several limitations. The pronouncement of death may have been delayed for minutes or longer after the terminal catastrophic event leading to death and the actual death due to logistic and individual disease factors. The time lag associated with discovering the cessation of vital signs, summoning the doctor, and performing procedures to certify death may vary among different hospital settings. This contributes to the uncertainty in the correlation between the recorded and actual death time. This problem is less prominent in hospitalized death cases than in-home death cases because in a hospital setting, the more frequent availability of staff and more readily available medical procedures shorten delays in the detection of a patient’s death.

Additionally, active resuscitation in the hospital may have been performed upon deterioration of the cancer patient’s condition, contributing to the patient’s prolonged survival. Attempts to resuscitate may occur more often in patients with sudden unexpected deterioration. The proportion of patients who died after attempted resuscitation is unclear. Furthermore, the accuracy of reported causes of death may vary with respect to whether an autopsy was performed and whether comorbidities were present before death. Delay in death reporting could decrease the peaks and increase the troughs of the observed temporal pattern of death, thereby concealing the actual temporal pattern of death.

Therefore, we consider the slightly greater number of cancer deaths at 6:00–6:59 am and at 8:00–8:59 pm to be an incidental finding related to healthcare delivery rather than to a chronobiological rhythm. However, owing to the retrospective population-based nature of the present study, the circumstances around individual patients could not be clarified. Finally, our findings did not rule out the possibility that the presence of external factors could be confounding for the circadian rhythm of death. However, data from a population database preclude detailed analysis of circumstances of individual patients. Future prospective studies should be conducted to further assess whether any external factors can influence the temporal pattern of death.

To conclude, we found no evidence of a chronobiological circadian pattern in death among cancer patients by using robust statistical methods and data from a large population in a hospital setting. Increased understanding of the temporal pattern of deaths may yield important insights toward understanding external factors associated with death.

## Additional files

**Additional file 1.** Materials and method, and model specification and formulae.

**Additional file 2: Figure S1.** Plot of the distribution of the prevalence ratios of death due to cancer, ischemic heart disease, and pneumonia by time (hour) in a day. 0:00–0:59 am is the reference hour.

**Additional file 3: Figure S2.** Scatter plot of the number of deaths by time in hours (A) and in minutes of the day (B), showing the temporal pattern of death due to ischemic heart disease, Hong Kong, 2008–2016. We found evidence of a unimodal sinusoidal circadian rhythm (periodicity) in the time of cardiac deaths according to the parametric sinusoidal circadian test (*Z* = 3.97, *P* = 0.019). Note: Restricted cubic splines using 3 knots were fitted to model the number of deaths in each hour of the day. The resulting spline fit is graphed as a red line.

**Additional file 4: Figure S3.** Scatter plot of the number of deaths by time in hours (A) and in minutes of the day (B), showing the temporal pattern of death due to pneumonia, Hong Kong, 2008–2016. We found no evidence of a unimodal sinusoidal circadian rhythm (periodicity) in the time of pneumonia deaths according to the parametric sinusoidal circadian test (*Z* = 1.94, *P *= 0.144). Note: Restricted cubic splines using 3 knots were fitted to model the number of deaths in each hour of the day. The resulting spline fit is graphed as a red line.

## Data Availability

The datasets generated and analyzed during the current study are available in the GitHub repository, https://github.com/bestmic/circadian_death/.
