# Supplementary material for: Is cancer-related death associated with circadian rhythm?
Source: Cancer Commun (Lond). 2019 May 14;39:27. doi: 10.1186/s40880-019-0373-9 (PMC6518786; doi:10.1186/s40880-019-0373-9)
Supplement: Supplementary file 1 — Additional file 1. Materials and method, and model specification and formulae. [file 40880_2019_373_MOESM1_ESM.docx]

**Additional** **Materials and Methods**

Data collection

Data were retrieved from the Clinical Data Analysis and Reporting System (CDARS), which is an electronic database operated by the Hospital Authority of Hong Kong. CDARS was established in 1995 for clinical audit and research purposes. The Hospital Authority is the sole public healthcare provider for primary, secondary, and tertiary care and covers approximately 90% of all secondary and tertiary care in Hong Kong, which has a population of approximately 7.4 million [1]. Data, including demographics, disease diagnoses, hospitalizations, and causes, times, and dates of death, are recorded in CDARS. The International Classification of Diseases, Ninth Revision (ICD-9), was used for disease coding. Positive and negative predictive values exceeding 90% have been demonstrated in previous studies [2, 3]. Patients’ privacy and confidentiality were protected by unique anonymous patient identifiers, which are linked to all the data contained in CDARS. Several high-quality population-based studies have been conducted based on the data retrieved from CDARS [3-5].

Study Subjects

We identified all adult cancer patients who were at least 18 years old and who were admitted and died in cancer palliative wards or acute oncology wards in all public hospitals in Hong Kong between January 2008 and December 2016. We included all the cancers in the list of ICD-9 (140 to 239: neoplasms). We also retrieved data on death due to ischemic heart disease (ICD-9 410-414) or pneumonia (ICD-9 486) in the same time period as in-patients in Hong Kong to compare the temporal pattern of death with cancer.

Statistical Analyses

First, we aggregated the number of deaths over a 60-minute period within 24 h. We then plotted the graph showing the temporal distribution of deaths and analyzed the relative change in the number of deaths during the time of a day in two different scales, i.e., hours and minutes. Restricted cubic splines were fitted using 3 knots to model the number of deaths. We calculated the prevalence (the number of deaths in each hour of a day divided by the total number of deaths at that hour of the day, over a period of 24 h) and the prevalence ratios between different hours of the day, always taking 0:00-0:59 am as the reference hour. The estimated prevalence ratios and their respective 95% confidence intervals (CIs) were derived using a negative binomial regression analysis accounting for over-dispersion [6]. We adjusted for age and sex, but not for cancer stage which could not be accurately retrieved for many cancer types. Prevalence ratios of age and sex were also presented.

To test the presence of a periodicity in a 24-hour period, we fitted a cosinor model, where the dependent variable death time was modeled as a sine wave characterized by a phase shift (i.e., location of peak and trough on the time axis) and amplitude (i.e., maximum variation of the sine wave from its mean height) [7]. The time variable (*t*) was measured on a discrete 24-hour scale and was transformed as cosine(*t*) and sine(*t*), which were then fitted as predictors of death. We derived a circadian periodic test using the transformed sine and cosine parameters for the cosinor model [7]. Model specification and formulae are also shown in the Appendix. The parametric sinusoidal circadian test assumes a 24-hour frequency characterized for being of sinusoidal pattern, unimodal in shape (i.e., the pattern occurs once per day), and the rhythm being the same each day (i.e., stationary). Finally, we used a non-parametric test of multimodality aiming to validate the results from the parametric statistical approach (cosinor model) and to evaluate whether the observed data support the presence of one unique mode (periodicity). A peak (or mode) was presented where an excess of probability mass (i.e., excess mass) was concentrated. By assessing the excess mass, we can statistically identify the number of peaks (or modes) presented. The null hypothesis of the non-parametric multimode test was that there was only unimodal circadian rhythm while the alternative supported the presence of more than one mode. A significant *P* value (< 0.05) rejects the presence of only one mode and therefore rejects the presence of a unimodal circadian pattern [8, 9]. Statistical analyses were performed using Stata v.15 (StataCorp, College Station, TX, USA) and R v. 3.5.0 (R Foundation for Statistical Computing, Vienna, Austria).

**Model Specification and Formulae**

Model specification was Eq. (A.1):

g(Y_t_) = c cos (w_t_) + s sin (w_t_), *t* = 1, … ,24

g(⋅) = link = log (number of cases); family: Negative Binomial.

where the frequency of the time of a day was converted to a circular scale Eq. (A.2):

w_t_ = 2π [hours_t_ − 0.5) / 24] k, k = Number of cycles per day.

where the amplitude (A) was defined as Eq. (A.3):

A = $\sqrt{c^{2}+s^{2}}$, (A ≥ 0).

and the phase (P) in time scale was Eq. (A.4):

P = 24 [(arctan(s/c) / 2π] + 1.

The circadian test statistic used was Eq. (A.5):

Z = nĀ^2^, Ā = $\sqrt{c^{2}+s^{2}}$.

The *P* value for the null hypothesis that Ā (average amplitude) = 0 was computed as the exponent of (-Z).

**References**

1. **Hospital authority statistical report 2016-2017** [http://www.ha.org.hk/haho/ho/stat/HASR16_17.pdf]

2. Wong OF, Ho PL, Lam SK: **Retrospective review of clinical presentations, microbiology, and outcomes of patients with psoas abscess**. *Hong Kong Med J* 2013, **19**(5):416-423.

3. Chan EW, Lau WC, Leung WK, Mok MT, He Y, Tong TS, Wong IC: **Prevention of Dabigatran-Related Gastrointestinal Bleeding With Gastroprotective Agents: A Population-Based Study**. *Gastroenterology* 2015, **149**(3):586-595.e583.

4. Chiu SS, Lau YL, Chan KH, Wong WH, Peiris JS: **Influenza-related hospitalizations among children in Hong Kong**. *N Engl J Med* 2002, **347**(26):2097-2103.

5. Cheung KS, Seto WK, Fung J, Lai CL, Yuen MF: **Epidemiology and Natural History of Primary Biliary Cholangitis in the Chinese: A Territory-Based Study in Hong Kong between 2000 and 2015**. *Clinical and translational gastroenterology* 2017, **8**(8):e116.

6. Hilbe JM: **Negative Binomial Regression**: Cambridge University Press; 2011.

7. Fisher NI: **Statistical Analysis of Circular Data**: Cambridge University Press; 1995.

8. Ameijeiras-Alonso J, Crujeiras RM, Rodríguez-Casal A: **Mode testing, critical bandwidth and excess mass**. *TEST* 2018.

9. Ameijeiras-Alonso J, Crujeiras R, Casal A: **Multimode: An R Package for Mode Assessment**. *arXiv* 2018, **1803.00472**
