# Supplementary figures and images for: Is cancer-related death associated with circadian rhythm?
Source: Cancer Commun (Lond). 2019 May 14;39:27. doi: 10.1186/s40880-019-0373-9 (PMC6518786; doi:10.1186/s40880-019-0373-9)

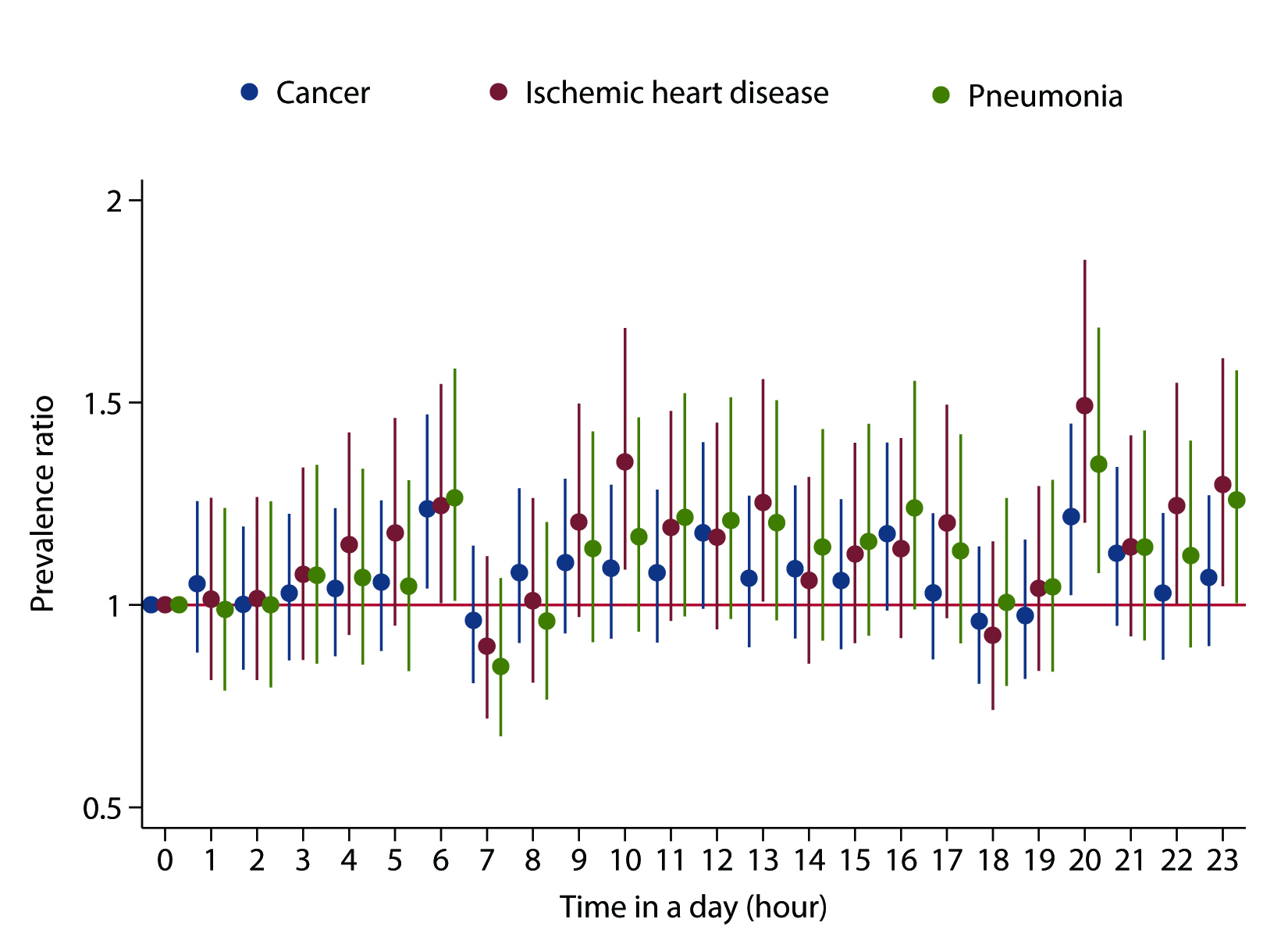

Supplement: Supplementary file 2 — Additional file 2: Figure S1. Plot of the distribution of the prevalence ratios of death due to cancer, ischemic heart disease, and pneumonia by time (hour) in a day. 0:00–0:59 am is the reference hour. [file 40880_2019_373_MOESM2_ESM.jpg]

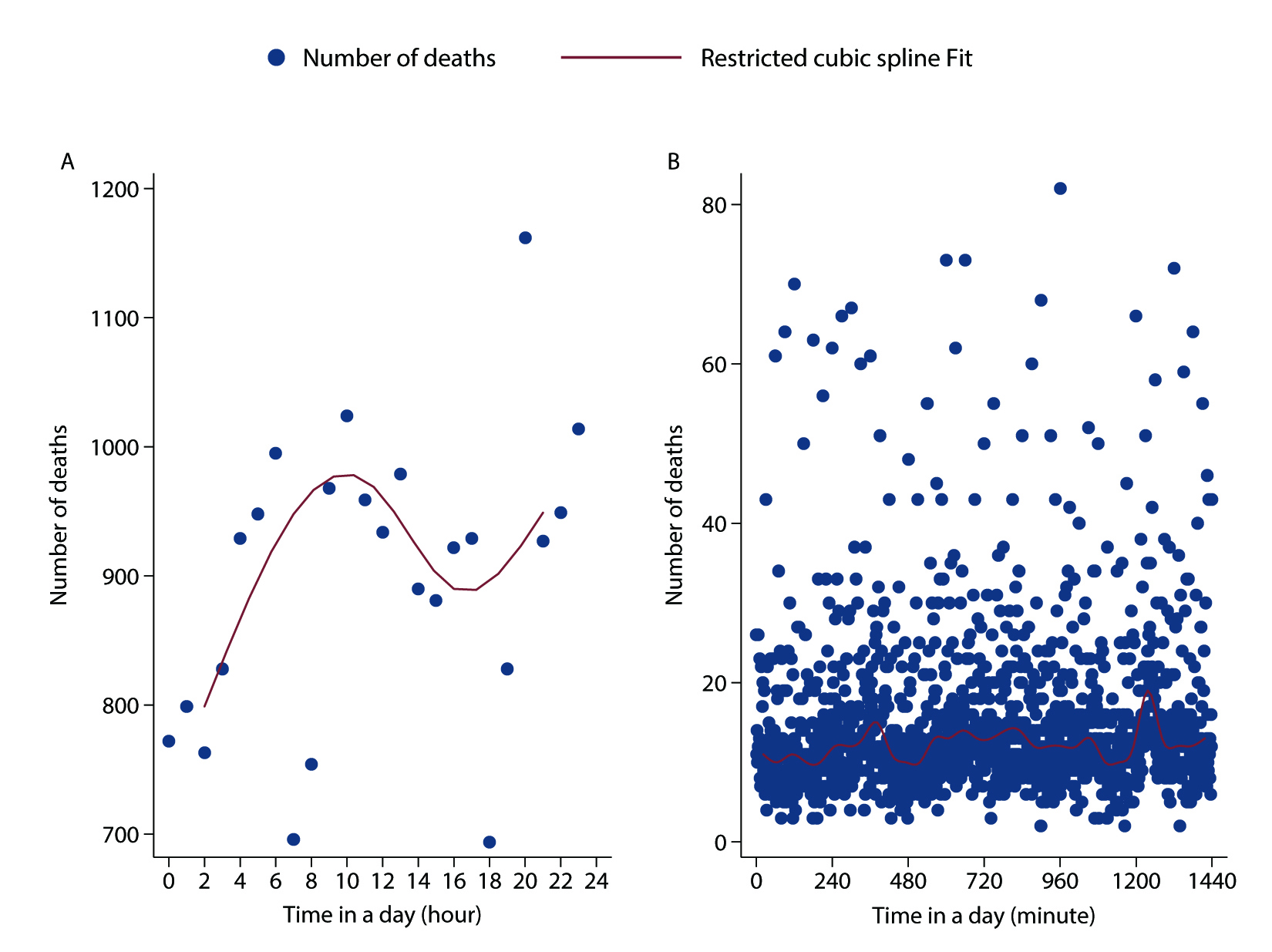

Supplement: Supplementary file 3 — Additional file 3: Figure S2. Scatter plot of the number of deaths by time in hours (A) and in minutes of the day (B), showing the temporal pattern of death due to ischemic heart disease, Hong Kong, 2008–2016. We found evidence of a unimodal sinusoidal circadian rhythm (periodicity) in the time of cardiac deaths according to the parametric sinusoidal circadian test (Z = 3.97, P = 0.019). Note: Restricted cubic splines using 3 knots were fitted to model the number of deaths in each hour of the day. The resulting spline fit is graphed as a red line. [file 40880_2019_373_MOESM3_ESM.jpg]

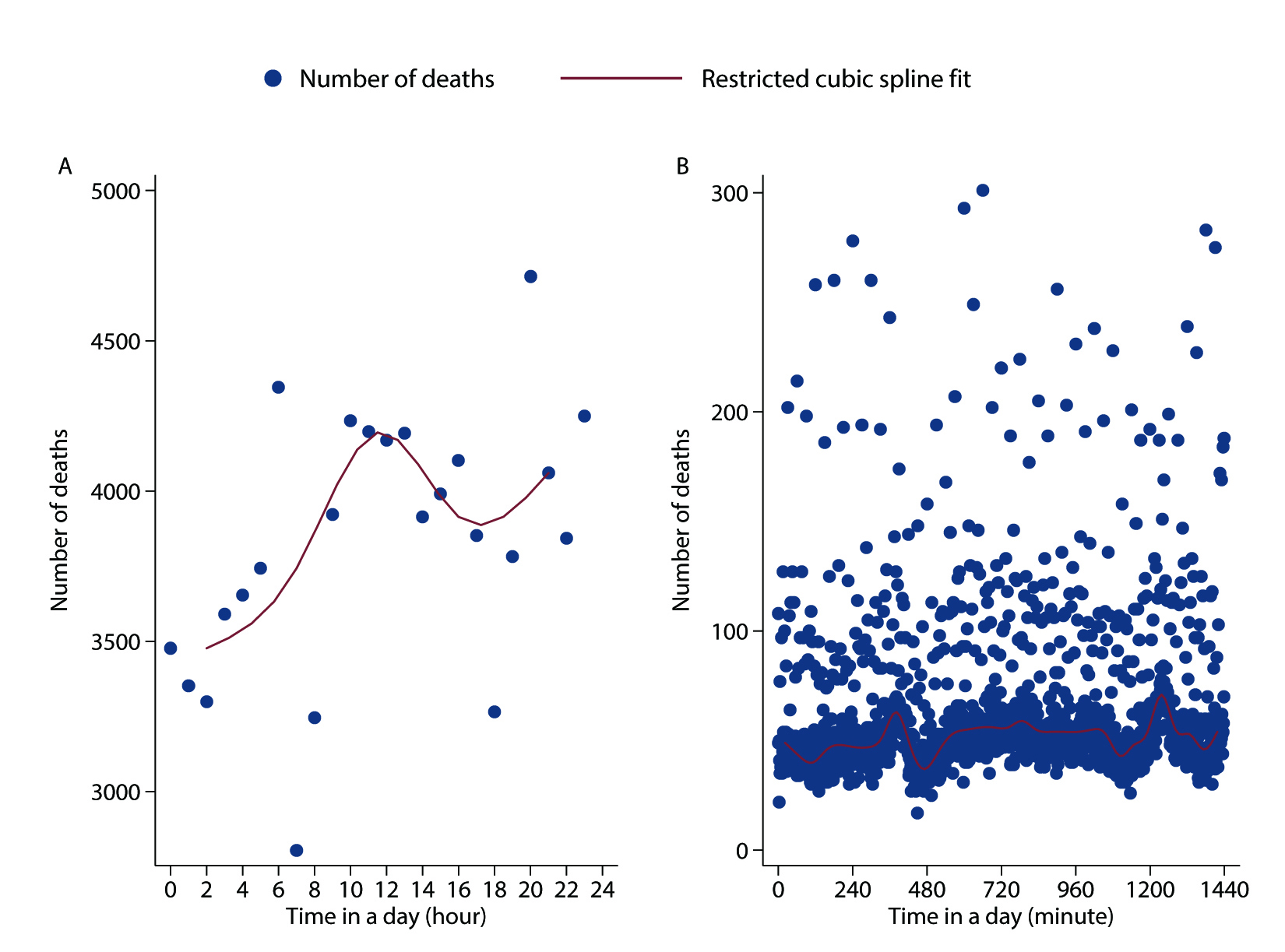

Supplement: Supplementary file 4 — Additional file 4: Figure S3. Scatter plot of the number of deaths by time in hours (A) and in minutes of the day (B), showing the temporal pattern of death due to pneumonia, Hong Kong, 2008–2016. We found no evidence of a unimodal sinusoidal circadian rhythm (periodicity) in the time of pneumonia deaths according to the parametric sinusoidal circadian test (Z = 1.94, P = 0.144). Note: Restricted cubic splines using 3 knots were fitted to model the number of deaths in each hour of the day. The resulting spline fit is graphed as a red line. [file 40880_2019_373_MOESM4_ESM.jpg]
